# Atopic Dermatitis-Related Problems in Daily Life, Goals of Therapy and Deciding Factors for Systemic Therapy: A Review

**DOI:** 10.3390/ph17111455

**Published:** 2024-10-30

**Authors:** Liborija Lugović-Mihić, Ema Barac, Renata Tomašević, Ena Parać, Lucija Zanze, Ana Ljevar, Lorena Dolački, Maja Štrajtenberger

**Affiliations:** 1 Department of Dermatovenereology, University Hospital Center Sestre Milosrdnice, 10000 Zagreb, Croatia; ema.barac@gmail.com (E.B.); renatavalo@gmail.com (R.T.); dolacki.lorena@gmail.com (L.D.); 2 School of Dental Medicine, University of Zagreb, 10000 Zagreb, Croatia; dr.strajs@gmail.com; 3 Family Physician Office, 10000 Zagreb, Croatia; lucijazanze15@gmail.com (L.Z.); ana.birt4@gmail.com (A.L.); 4 Division of Pulmonology, Immunology, Allergology, and Rheumatology, Department of Paediatrics, University Children’s Hospital, 10000 Zagreb, Croatia; enaparac7@gmail.com; 5 Department of Pulmonology, Special Hospital for Pulmonary Diseases, 10000 Zagreb, Croatia

**Keywords:** atopic dermatitis, treatment, quality of life, disease burden, therapy, economic costs, psychological aspects, daily activities, JAK inhibitors, biologics, dupilumab, atopic eczema

## Abstract

**Background/Objectives/Methods**: Atopic dermatitis (AD) impacts various aspects of patients’ lives including personal life, psychological aspects/disturbances (e.g., depression, anxiety, or even suicidal thoughts), school, and work-related activities, including career advancement. The aim of this narrative review is to present the latest information available on how to best approach AD patient management, as well as decisions regarding standard/advanced systemic therapy, by gathering evidence from the relevant medical literature (PubMed and other prominent medical databases). **Results**: Thus, AD patient management and decisions regarding advanced/systemic therapy are complex, requiring the consideration of multiple disease-related factors: age; disease severity; patient medical history and comorbidities; previous topical therapy use and any adverse reactions; treatment efficacy concerns; patient preferences, expectations and fears; pregnancy planning; ability and willingness to adhere to the treatment regimen; impact on related risks; and any associated psychological or psychiatric issues. Current guidelines and systematic reviews support the safety and efficacy of systemic therapy including conventional drugs (cyclosporine, methotrexate, and azathioprine), biologics (dupilumab and tralokinumab), and JAK inhibitors (baricitinib, upadacitinib, and abrocitinib) recommended for treating moderate and severe AD. Recently, additional biologics have been evaluated in clinical trials, including lebrikizumab, nemolizumab, eblasakimab, and OX40/OX40L, among others. **Conclusions**: The most recently suggested approach to treating AD patients suggests focusing on therapy that targets and achieves minimal disease activity (MDA), where therapy decisions are informed by both the patient and the clinician. Available data also indicate the importance of a personalized, stepwise, and multidisciplinary approach. This type of approach promotes patient compliance, satisfaction with therapy, and increased engagement, which all lead to better patient outcomes.

## 1. Introduction

**Figure 1:**
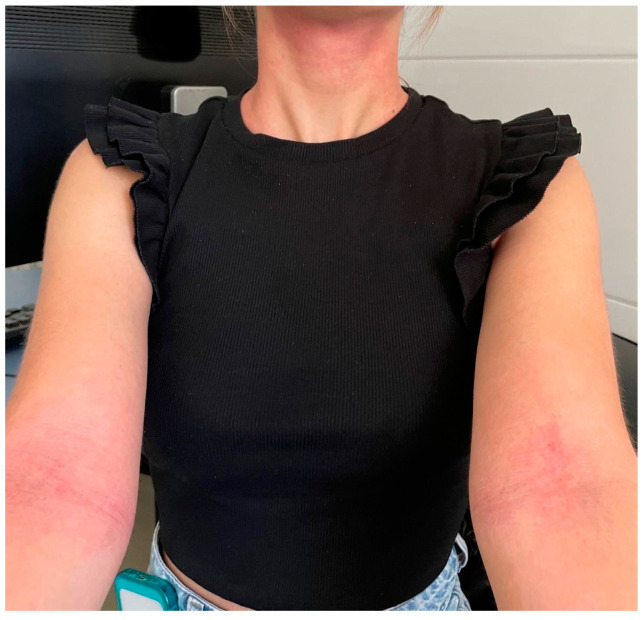
Common skin manifestation of atopic dermatitis in adults with lesions on flexural sites and the neck (image from the archive of the main author, Prof. L. Lugović-Mihić, obtained with patient consent).

Atopic dermatitis (AD) is a chronic skin disease that often impacts emotional well-being, work productivity, daily activities, and more. The most common manifestations of AD include eczematous skin lesions, dryness, and itching, along with impaired sleeping. Clinically, AD is a dermatosis characterized by episodes of acute flare-ups and remissions, with symptoms that can vary [1,2]. In the acute stage of AD, lesions are erythematous, sometimes accompanied by exudation, erosions, crusts, and scales. Gradually, chronic skin changes develop, often resulting in persistent lichenified lesions (Figure 1). Among the clinical features of AD, itching is most frequently reported, although its prevalence varies (ranging from 21% to 100%) [3,4,5,6]. The disease is more common in children but can occur at any age, presenting with various clinical pictures.

Not all aspects of the etiopathogenic processes underlying AD have been definitively established. Nevertheless, AD is recognized as a multifactorial disease with numerous etiological and contributing factors, including an impaired skin barrier, genetic predisposition, susceptibility to allergies, Th2-mediated immune responses, and decreased antimicrobial peptides, among others [1,2,7,8,9].

The quality of life for patients with AD is significantly affected by the disease, with the extent of the impact largely dependent on its severity. Additionally, AD imposes a significant burden on society, primarily due to its high prevalence, which has increased since the late 20th century. This prevalence, in the general population, is notably higher in children (up to 10–20%) compared to adults (3–5%) [10,11,12]. Also, since psychological changes such as depression and anxiety are also common in AD patients, it is important to consider various factors when approaching the disease, rather than focusing solely on skin lesions [13].

**Figure 2:**
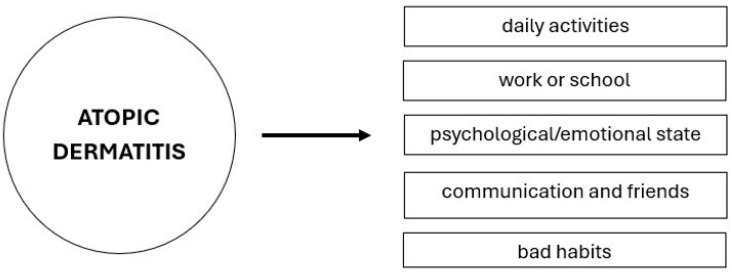
Areas of patient life affected by atopic dermatitis.

Since AD is non-fatal by nature, it is often neglected compared to more severe and life-threatening diseases. However, several studies indicate it carries with it an enormous burden due to its significant impact on wellbeing and consequent psychosocial effects [14,15,16]. Thus, it is necessary to consider not only the clinical aspects of AD but the economic and human cost of the disease as well, remembering that both adults and adolescents are affected [13]. Understanding the burden of AD on individuals and society is key for designing effective public health policies, including intervention priorities and resource allocation [17]. Therefore, it is essential to evaluate the burden of AD through scientific evidence to inform both doctors and patients about the disease and its characteristics (Figure 2). This can help decision-makers in developing treatment strategies.

Considering all of these aspects of AD, the purpose of this review is to present the latest information available on how to best approach AD patient management and decision-making regarding standard/advanced therapy. We aim to identify, present, and summarize previously published data on this topic, including information on AD patients’ problems related to their disease in daily life, goals of therapy, and deciding factors for systemic therapy. For this narrative review, we analysed literature data published in prominent medical databases during the period between 2013 and 2024. Thus, for data sources and search strategy, we included the PubMed, Web of Science (WOS) and Scopus databases. The search was carried out using the medical terms: “atopic dermatitis”; “treatment”; “quality of life”; “disease burden”; “therapy”; “economic costs”; “psychological aspects”; “daily activities”; “JAK inhibitors”; “biologics”; “dupilumab”; and “atopic eczema”. To broaden the search, we used synonyms as keywords like “atopic dermatitis” and “atopic eczema”, as well as “therapy” and “treatment”. Finally, in the last part of this review, we wanted to present key results from recent studies (worldwide) on AD therapy decisions and patient preferences. Due to word limits and the nature of this article, which is a narrative review, not a meta-analysis, we cited only key references. So, this analysis explores existing knowledge, based on previous studies conducted on this topic, and presents current data, with the aim to reach a logical organization structure. Thus, we present current data useful to understanding optimal/the best approaches to AD patient management and decision-making regarding standard/advanced therapy in clinical practice.

## 2. The Impact of Atopic Dermatitis on Emotional and Psychological States

Research indicates that the most reported factors affecting clinical burden are itching (pruritus) and psychological disturbances such as depression and anxiety (51% and 49%, respectively) [13]. Among the most significant impacts are its negative effect on sleep, primarily due to itching. This is supported by evidence of impaired sleeping indicators and decreased melatonin production [18,19]. While short-term sleep deprivation, such as a single night of poor sleep, is typically manageable, the impact of lack of sleep is significantly intensified in chronic diseases like AD. In such cases, sleep disorders occur in most patients. These disturbances can lead to additional issues, including reliance on sleeping pills, decreased concentration and lethargy [20].

In the past, AD was commonly known as “neurodermitis” due to the psychological features and changes observed in these patients, who were often perceived as neurotic [21]. This is because of the associated occurrence of anxiety and depression that significantly burdens AD patients compared to the general population. Numerous studies have reported a high prevalence of depression among patients with AD [3,22,23,24]. Research indicates that the prevalence of depression among these patients averages 18%, with a range from 3% to 57%. These findings align with self-reported data on depression, which average 26% (ranging from 10% to 37%) [25,26,27]. Moreover, the results of numerous studies show that the average prevalence of anxiety in AD patients is 24.12% (ranging from 1.2% to 64%).

A particularly important topic to consider here is stress, which can both contribute to and result from AD. Studies indicate that psychological stress is prevalent among patients with AD, with 46% experiencing high stress and 21% experiencing moderate stress [21,28,29]. Many AD patients report an association between recent stressful events and disease exacerbation, a finding supported by research/literature data [30]. Physically, the visibility and functional impact of AD skin lesions often lead to low levels of happiness and higher levels of anxiety, depression, and feelings of stress [31]. Negative social and psychological changes can also result in depression, anxiety, and even suicidal ideation (44%) and suicide attempts (36%) [29,32]. Physiologically, altered cortisol levels (as a marker of stress) have also been confirmed in AD patients. In the early phase of the disease, increased cortisol values were recorded (overactivated HPA axis). This stimulates skin neural structures, deteriorates the skin’s Th2 response, and reinforces habitual scratching. In the later stages of AD, there is a weaker HPA axis function and a shift to a Th1-dominant response [21,33]. When taking the above into account, addressing the impact of stress on patients, especially young individuals with AD, could be vital for improving their mental health [7].

According to research comparing patients with AD during exacerbation to those who are asymptomatic (in AD remission), the severity of AD (as measured by SCORAD values) positively correlates with impaired quality of life (measured by Dermatology Life Quality Index, DLQI) and the overall impact of the disease on daily life and symptom management [34]. Impaired quality of life (DLQI) is also associated with certain personality features (anxiety, obsession, depression, and somatization). Thus, when managing patients, it is important to consider not only the severity of AD but also how personality characteristics such as anxiety disorders, somatization, obsession, and depression affect quality of life.

There have also been observations that certain habits and behaviours, such as smoking and alcohol use, may be linked to increased stress and depression in AD patients [7]. In turn, these habits negatively impact AD, leading to a vicious circle. According to literature, smoking stimulates the surface of immune cells, increases the total number of white blood cells, and increases Th2 cells responsible for allergic immune responses and IL-4 (key in the treatment of chronic inflammation). Excessive alcohol consumption is also negatively associated with AD, as alcohol increases IgE production, which stimulates Th2 cells and exacerbates AD (like the effects of smoking). It also causes skin dryness and an acute inflammatory response due to histamine release from acetaldehyde [35,36,37]. Specific issues are also noted in sensitive populations such as adolescents who suffer from AD, who often struggle with body image due to chronic/recurrent inflammatory lesions. Thus, aesthetic and functional skin lesions lead to low happiness, high stress, and depression [3]. Additionally, smoking and alcohol consumption in adolescents with AD reduce immune function, which exacerbates the condition and increases stress and depressive symptoms. Overall, adolescents with AD may struggle with establishing a healthy body image and may experience significant social and psychological issues [29].

However, engaging in physical activities can help patients reduce the psychological stress that is often related to their AD. According to research results, those who engage in regular physical activity have a 30% lower risk of stress than those who do not. This is likely because physical activity helps reduce cortisol levels (secreted in significant amounts in response to stress) and helps produce and activate endorphins that directly affect the brain [38,39]. In addition to the emotional, psychological, and mental health issues associated with AD, social functioning problems ultimately arise [40]. Consequently, recent management guidelines for AD now emphasize the importance of addressing the psychological aspects of the disease, which may require psychosomatic counselling or sometimes even psychiatric drugs.

## 3. The Impact of Atopic Dermatitis on Daily Activities, Work Productivity, and Quality of Life

Patients with AD, particularly those with moderate to severe forms, often experience a noticeable impact on their effectiveness and productivity at work or school due to frequent exacerbations. Aside from typical AD lesions, itching, and sleep disturbances, individuals with AD also have an atopic constitution that predisposes them to contact dermatitis, often on the hands. This additional skin condition can further disrupt their productivity and affect their work performance [41,42,43]. AD significantly impacts productivity, with research showing 68.8 days lost annually due to absenteeism and productivity loss. Notably, productivity loss accounts for most of this impact, with 54 lost days, which is three times higher than absenteeism, which accounts for 14.8 lost days [44,45,46,47,48,49,50,51]. Productivity loss significantly depends on the severity of AD. According to literature data, patients with severe AD lost an average of 26.5 days due to absenteeism and 92.5 days due to productivity loss, while patients with mild AD lost an average of 2.5 days due to absenteeism and 13.6 days due to productivity loss [47,51].

Frequent doctor visits also have a significant impact on patients. According to studies, AD patients make between 2.8 to 16.3 visits to a dermatologist annually, with an average of 8.6 visits [46,52,53,54]. Additionally, visits to primary care or general practitioners average 16.5 annually and significantly depend on AD severity. For example, patients with moderate to severe AD had 20.44 annual visits to the doctor [52,54]. Furthermore, research shows that AD patients sometimes visit non-dermatological specialists, such as allergy and internal medicine specialists (with a rate of 0.2–0.4 visits annually) [46,52]. As the severity of AD increases, visits to the emergency department become more frequent, although admissions to the emergency room remain rare [53,54,55,56,57]. According to previous study results, the average annual number of emergency department visits varies: 0.5 per patient for milder cases (grade 2 severity), 0.92 visits for patients with grade 3 severity, and 1.41 visits for those with grade 4 severity [53,54,56]. The average annual number of hospitalizations ranged from 0.03 to 1.2 admissions—more frequent for patients with grade 4 severity (0.75 per year) than those with grade 2 severity (0.45 per year) [53,56,57].

## 4. Economic Costs of Atopic Dermatitis

The treatment of AD imposes a significant financial burden due to the disease’s prevalence and chronic nature. Indirect costs, such as a loss of productivity, often exceed direct treatment costs. There is notable variability in cost estimates across studies from countries with different income levels. Based on multiple studies, the total cost of AD per patient, expressed as an average annual cost, is estimated at USD 5246 (2020), with a range from USD 769 to USD 23,638 [46,47,54,58]. This average total cost is lower than the combined average of direct and indirect costs due to differences in data sources and calculation methods. Numerous studies have reported that the average annual direct costs reach USD 4411 [46,59,60], while the average annual indirect costs have been reported in three studies and equal USD 9068 [46].

Several studies on AD confirm significant reductions in patient quality of life and increased school or work absenteeism due to AD [13,61]. Findings on this topic vary across the literature. Drucker et al. estimated the total annual cost per patient in the U.S. to range from USD 3302 to USD 4463, while Fasseeh et al. estimated it at USD 4411 (which is not limited to the U.S.) [13,57]. The burden of AD might be underestimated in low- and middle-income countries as, despite the abundance of literature on this subject, most research comes from high-income countries (low- and middle-income countries were not equally represented in the literature). A study on the global burden of disease found a positive correlation between the burden of AD and gross domestic product; however, this may be due to insufficient data and the underreporting of AD in low- and middle-income countries [12].

## 5. Standard Therapy and Advanced Treatment Possibilities

The treatment of AD has significantly advanced, with regulatory institutions worldwide approving new drugs for AD patients, while other medications remain in various stages of clinical trials [24,62,63,64]. Current AD therapy includes routine skincare, the avoidance of triggers, topical and systemic treatment, and other measures. According to current global recommendations and guidelines, the treatment of AD primarily depends on the severity of the disease [24,62,63,64]. The 2018 guidelines classify AD severity into mild, moderate, and severe based on the SCORAD index/questionnaire (SCORAD > 50 or persistent eczema is severe; SCORAD 25–50 or recurrent eczema is moderate; and SCORAD < 25 or transient eczema is mild).

However, regardless of severity, the primary treatment for all forms involves patient and family education, regular use of emollients and oil baths, and the identification and avoidance of environmental factors (non-specific irritants and specific allergens) that exacerbate the disease [62]. Typical topical AD therapy primarily involves topical corticosteroids, which are generally the most effective at controlling skin lesions [2,63,64]. However, these should be used for relatively short periods due to potential adverse effects such as skin atrophy, rebound phenomenon, telangiectasias, striae, folliculitis, purpura, contact dermatitis, adrenal suppression, etc. [2,65]. It is known that “corticophobia” is frequent among AD patients, therefore, when lesions persist, topical immunomodulators are recommended (tacrolimus and pimecrolimus) [2]. Another topical option is topical crisaborole, a boron-based phosphodiesterase 4 inhibitor (PDE-4), which inhibits overactive PDE-4 enzymes contributing to AD manifestations [2,65].

In addition, phototherapy, including UVB 311 nm, and occasionally UVA1 and PUVA, can be used for severe forms of AD in adults. Phototherapy is mainly administered two to three times per week.

When topical therapy and phototherapy are insufficient for the management of AD, there is a need for conventional systemic therapies (cyclosporine, methotrexate, and azathioprine) or alternative systemic treatments, such as biologics and Janus kinase (JAK) inhibitors [2,62]. Conventional systemic drugs for AD include certain immunosuppressants that calm skin inflammation [2]. Among systemic medications for AD, only cyclosporine has been approved for AD, while azathioprine, methotrexate, and mycophenolate mofetil are prescribed off-label [66,67]. Cyclosporine is an immunosuppressant that inhibits various immune cells (like T cells, NK cells, antigen presentation by APCs, and the production of IL-2 and GM-CSF). However, while adverse reactions are multiple, they are relatively uncommon and include nephrotoxic effects, hypertension, changed blood counts, gingival hyperplasia, hypertrichosis, headaches, etc.) [67]. Methotrexate is an immunosuppressant that inhibits the synthesis of cell structures (DNA, RNA, and purines), thereby inhibiting T cells. Its side effects include hepatotoxicity, leukopenia, thrombocytopenia, etc. It is available in oral and subcutaneous forms and is typically taken with folic acid to mitigate adverse effects. Azathioprine, a purine analogue, blocks DNA synthesis in T and B cells. Its potential side effects include hepatotoxicity, gastrointestinal symptoms, leukopenia, etc. Mycophenolate mofetil is an immunosuppressant drug used to treat various autoimmune and inflammatory conditions. Its adverse effects include nausea, diarrhoea, infections, and other complications [68].

Recently, several drugs have been approved for moderate to severe AD, including biological drugs that predominantly inhibit interleukins (IL-4, IL-13, and IL-33) [2,62]. Dupilumab is a biologic that blocks IL-4 and IL-13 receptors and is indicated for patients with moderate to severe AD resistant to standard therapy. Though side effects are infrequent, they can include ocular complications, conjunctivitis, injection site reactions, etc. [21,69]. Tralokinumab is another biological agent, a monoclonal anti-IL-13 antibody, also indicated for moderate to severe AD unresponsive to topical preparations [70]. Additional biologics being evaluated in clinical trials include lebrikizumab, nemolizumab, eblasakimab, OX40/OX40L, and others.

In addition to systemic therapies previously mentioned, three JAK inhibitors—baricitinib, upadacitinib, and abrocitinib—have been approved for moderate to severe AD. There is limited data available on the effectiveness of other JAK inhibitors (ruxolitinib, tofacitinib, and deucravacitinib) [2,71]. JAK inhibitors affect intracellular transducing signals, which in turn influence blood cell formation and the functioning of the immune system. JAKs phosphorylate and activate cell transcription, which modulates intracellular processes, including genetic expression. Nowadays, JAK inhibitors are among the best treatment options for severe AD due to their effectiveness and safety [2,72,73,74]. Based on real-life data results, they provide rapid improvement in AD symptoms (especially itching), leading to an improved patient quality of life. Although the safety profile is favourable, long-term safety data are still limited. Potential side effects include upper respiratory tract infections, increased blood lipids, nausea and abdominal pain, herpes virus infections, acne, creatine kinase elevation, thrombocytosis, headache, etc. [2]. One study (meta-analysis) by Silverberg et al. compared the effectiveness of targeted systemic therapies for the treatment of moderate to severe AD [24,65]. According to their results, upadacitinib 30 mg daily, upadacitinib 15 mg daily, and abrocitinib 200 mg daily were the most effective targeted systemic therapies during their follow-up (12 to 16 weeks of AD treatment) [24,71].

However, when approaching patient care, it is important to note that therapy depends on the specific form of AD and the severity of the disease. For mild forms of AD, in addition to skin care, topical anti-inflammatory therapy (class II topical glucocorticoids) is recommended during periods of exacerbation [62,64]. If necessary, as in the case of secondary infection, treatment with antiseptic compresses or silver-infused wraps may be advised. For persistent AD lesions and specific areas (e.g., lesions on the face, neck, folds, and anogenital region), topical calcineurin inhibitors (tacrolimus ointment and pimecrolimus cream, approved for use in patients aged two years and older) are recommended as the therapy of choice. Systemic antibiotic therapy is recommended only in cases of extensive superinfections.

For moderate forms of AD, in addition to primary treatment, proactive therapy with topical tacrolimus or class II or III topical glucocorticoids is advised (e.g., for glucocorticoids twice weekly for up to 16 weeks, and calcineurin inhibitors for up to 52 weeks). For chronic lichenified lesions in AD that recur despite topical therapy, phototherapy (such as UVB) is advised. However, prior evaluation for potential contraindications and photosensitivity is needed [62,63,64]. For severe forms of AD where disease control is not achieved through primary treatment measures, topical therapies, phototherapy, or systemic immunosuppressive therapy are recommended for adults. Options include cyclosporine A (for 3 to 6 months), short-term oral glucocorticoids (up to 7 days), mycophenolate mofetil, and methotrexate. Phototherapy such as PUVA (psoralen and UVA), or alitretinoin (for isolated hand eczema) may also be considered. For moderate to severe forms of AD that do not respond to topical treatments and/or phototherapy and where systemic conventional therapy is not recommended, biological therapy is indicated. The first line of biological therapy is dupilumab, a monoclonal antibody that blocks IL-4 and IL-13 receptors; nowadays, JAK inhibitors also exhibit similar excellent effects [69,72,73,74]. Additionally, psychological support and alternative treatment methods can also be considered [62].

**Figure 3:**
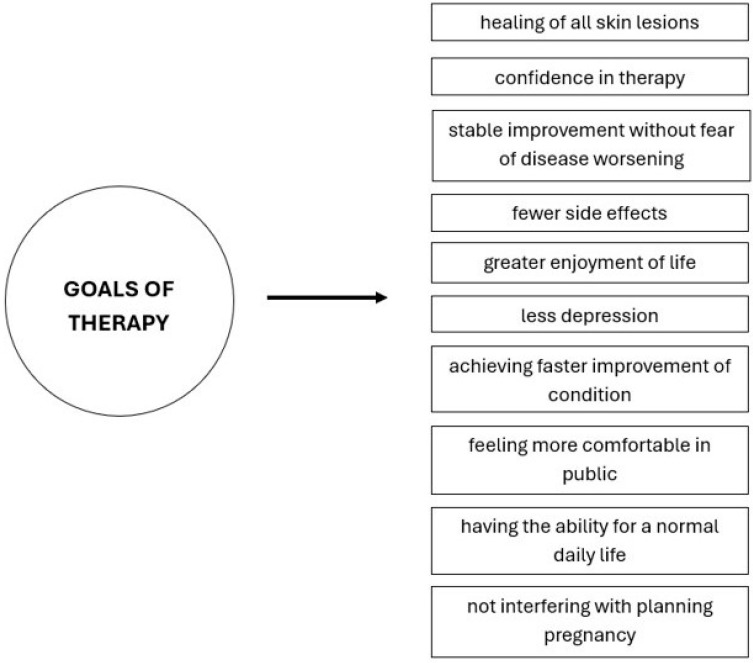
Goals of therapy for atopic dermatitis.

Most AD patients have mild to moderate disease, which is usually well managed with emollients, standard topical anti-inflammatory preparations, and by avoiding triggers. However, a significant number of patients still do not achieve adequate control with this therapy [75]. For patients with moderate to severe AD who do not respond to topical therapy and where phototherapy is not suitable, systemic treatment is necessary to control the disease, reduce symptoms, prevent relapses, and improve quality of life (Figure 3).

The latest data from the literature points to patient opinion and preference as being very important factors regarding therapy options and final therapy decisions. Thus, it is necessary to consider the patient’s individual characteristics, such as age, gender, disease duration, and many other factors. It should be noted that there is sometimes a poor correlation between the patient’s condition, needs, and quality of life (DLQI). This discrepancy may suggest that a patient’s needs can differ significantly from their perceived burden of disease and quality of life, highlighting the need for additional evaluation.

## 6. Achieving Therapeutic Goals

Key factors in achieving a patient’s therapy goals are, predominantly, the patient’s own insight into their condition, the clinician’s scoring/index of the condition, and the patient’s disease-related problems and therapy preferences. Nowadays, there are different advanced therapy options [73,74,76]. Guidelines and systematic reviews from national associations provide evidence of the safety and efficacy of systemic therapy (conventional, biologics, and JAK inhibitors) for treating AD [56,62,73,74,77]. Thus, according to one study that examined AD patients’ needs related to therapeutic goals (through a comprehensive survey of 1678 AD patients), their most crucial therapeutic goal was to reduce itching, even more important than healing all skin lesions [4]. However, many other AD-related needs are also essential, including improving perceived quality of life, as results show that a patient’s experience significantly reduced quality of life (confirmed by DLQI scores). Other important factors are gender, age, and duration of disease. Although no significant differences in quality of life were found between men and women, significant differences were observed across different ages, e.g., older adults reported significantly lower quality of life compared to younger adults. General health levels were also lower in older adults than in younger adults, likely due to factors associated with ageing, such as reduced mobility and increased difficulties in daily activities.

Considering the impact of AD duration on patients, those with a shorter duration of AD (up to one year) experience greater impairment in quality of life compared to those with a longer duration (over one year). The difference is likely because newer patients have not yet adapted to the disease or developed effective coping strategies [4,13]. Different findings have been observed regarding the impact of disease duration on AD patients. According to a study by Augustin et al., patients with a shorter AD duration (up to one year) expressed a greater need for reassurance that the disease will not worsen, for healing all skin lesions, for a clear diagnosis and effective therapy, and for confidence in their treatment (i.e., those with shorter disease duration experienced a more significant burden) [4]. However, study results differ, as previous research had found that patients with longer disease duration may experience a more significant overall burden. It is also necessary to consider the patient’s individual characteristics, such as age, gender, disease duration, and many other factors. Occasionally, the patient’s needs poorly correlated with their quality of life (DLQI), which may indicate that patients’ needs can differ significantly from their perceived burden of the disease and quality of life, highlighting the need for additional evaluation.

Studies on the needs of AD patients and their therapy decisions indicate various disease-related requirements that significantly differ by age, gender, and disease duration, making it more critical for older patients to achieve therapeutic goals compared to younger individuals [73,74,76]. This is particularly evident in their need for fewer side effects, greater enjoyment of life, confidence in therapy, and reduced depression (based on surveys) [4]. However, the ability to lead a normal sexual life is more important for younger adults than for older ones, as anticipated. In terms of gender, women placed greater emphasis on achieving faster improvement in skin appearance, feeling more comfortable in public, healing all skin lesions, maintaining a normal daily life, and not fearing disease progression [4].

**Figure 4:**
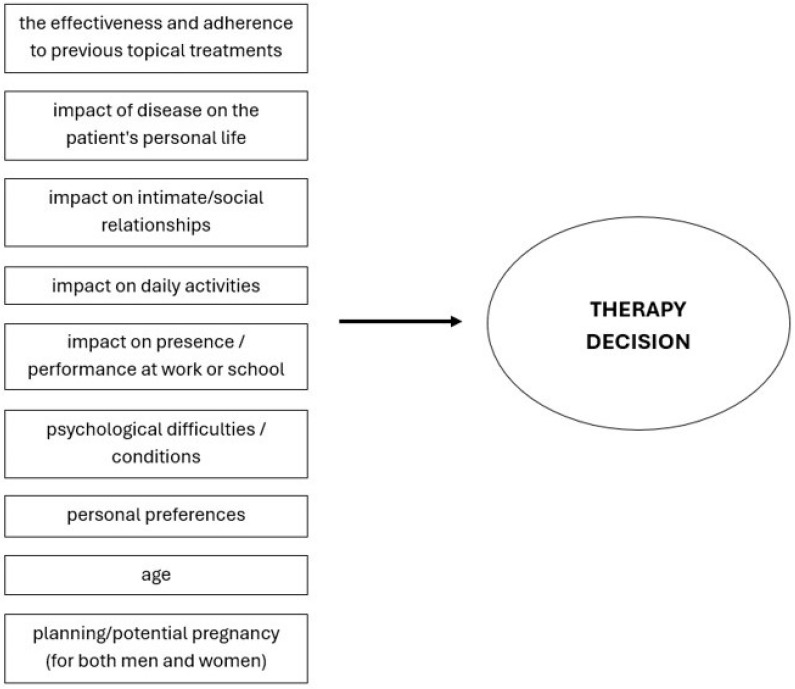
Factors influencing the decision over systemic therapy for atopic dermatitis.

Overall, greater needs for AD treatment are observed with older age, more severe disease, and greater impairment in quality of life, with the extent of impairment correlating with an increased need for treatment. The most recent approach to treating patients with AD is focused on therapy that targets and achieves minimal disease activity (MDA), where the decision on therapy is based on both patient and clinician decisions [65]. Also very important is understanding and taking into account patients’ preferences. This shared patient–clinician approach may improve disease management/outcome through better patient compliance, satisfaction with therapy, and increased engagement [78]. So, in clinical practice, it is recommended that both physicians and patients collaborate in advance to identify specific needs and potential therapeutic goals, leading to an individualized and personalized therapeutic decision through a shared decision-making process and a multidisciplinary approach [4]. Thus, clinicians and patients (or caregivers) should consider multiple factors when deciding whether and when to introduce systemic therapy (Figure 4). While this shared decision-making approach is the currently accepted best approach to the management of AD, universally accepted criteria for it have not yet been defined [77].

## 7. Decision to Initiate Systemic Therapy

When approaching an AD patient who may be a candidate for systemic therapy, it is necessary to determine the severity of the disease using scoring scales that quantify AD severity (there are over 20 such scales available) [2,62,77]. The two most used scoring scales are the Scoring of Atopic Dermatitis (SCORAD) and the Eczema Area and Severity Index (EASI). The SCORAD scale assesses the intensity of disease manifestations, the extent of affected areas, sleep loss, and itching. The EASI score, on the other hand, provides a more comprehensive evaluation by incorporating additional clinical data. However, quantifying the severity of AD is often challenging because the lesions are sometimes diffuse and poorly defined. Many European dermatologists, for instance, prefer using a SCORAD > 25 to categorize the disease as moderate to severe. However, relying on a single scoring scale has several drawbacks given the characteristic flares and remissions of AD. Multiple assessments are often needed to accurately reflect the baseline state of the disease, flares, and therapeutic responses. It is also important to evaluate the severity and frequency of AD flares using various methods, taking into account their impact on quality of life and the effectiveness of current therapy. Self-assessment scales, such as the Patient-Oriented Eczema Measure (POEM) and the Patient-Oriented SCORAD, can also be useful. These scales reflect the patient’s perspective on the disease course between consultations. Repeated documentation of the presence of severe, extensive disease and/or significant impairment in quality of life (despite adequate topical therapy) can justify the transition to systemic therapy [13,77,78].

In addition to assessing the severity of the disease, it is also essential to consider the impact of the disease on their quality of life. Even localized AD affecting small areas (e.g., only the face, hands, or genital area) can significantly impact the patient’s emotional state, social interactions, and daily activities. However, scoring scales are time-consuming for routine clinical practices and assess only certain aspects of the disease, making them primarily useful in clinical trials [77]. Moreover, an assessment based solely on a scoring system cannot determine the need for systemic therapy; a comprehensive and holistic assessment is still required.

Nevertheless, when considering the introduction of systemic therapy for AD, it is crucial to carefully evaluate the known risks of conventional immunosuppressive treatments, such as the side effects of cyclosporine. Until recently, corticosteroid therapy was the most common systemic treatment for AD, with 10% of AD patients receiving this treatment, although it is less commonly employed in children with AD [77].

From a therapeutic perspective, the availability of targeted immunomodulation, such as JAK inhibitors and biologics, with lower risks to patient safety, facilitates the introduction of systemic therapies by lowering the threshold for its use [2,62,73,74,78,79,80]. Experience with several advanced therapies, when compared to conventional treatments, indicates that these newer options may offer superior efficacy and short-term safety. Registries are valuable for evaluating long-term safety and efficacy profiles, allowing comparisons between conventional immunosuppressive therapies and emerging new therapies. The threshold for introducing advanced therapies is often lower in moderate AD, aiming to improve disease response and quality of life, as well as to potentially prevent disease progression and future comorbidities [77].

So, before initiating systemic therapy for AD, it is necessary to consider several key factors that determine whether a patient is a suitable candidate for this treatment. Systemic therapy is usually introduced for patients who fail to respond to adequate topical therapy or who experience frequent relapses, often requiring the prolonged use of high-potency topical corticosteroids. In patients with moderate to severe forms of AD, several factors should be considered, including frequent recurrences of the disease, previous adverse effects of therapy, such as skin atrophy, and the effectiveness and adherence to previous topical treatments. It is also important to assess the impact of the disease on the patient’s personal life, including intimate aspects and pregnancy planning, social relationships, and engagement in activities. Additionally, the disease’s impact on work or school performance and the presence of concurrent AD-related psychological difficulties or relevant prior psychological disorders should be evaluated. During discussions with the patient, it is necessary to determine their willingness and ability to adhere to the treatment regimen, understand their expectations for the treatment, and their concerns about side effects. When choosing systemic therapy, it is important to integrate the patient’s medical history, treatment preferences, and risk factors associated with the therapy [68]. In addition, it is also important to consider associated factors such as atopic comorbidities, autoimmune diseases, and cardiometabolic disorders, along with behavioural disorders, associated neuropsychiatric comorbidities, and others [81]. Potential allergens and comorbidities (primarily allergic diseases, like allergic asthma, allergic rhinoconjunctivitis, oral allergy syndrome, food allergy, etc.) should also be considered and treated [82,83]. For example, AD patients are often allergic to inhaled allergens (type I reactions) or, occasionally, to contact allergens (delayed type IV allergic reactions) [2,82]. These conditions also should be considered when deciding on therapy for AD.

It is important that patients are informed in advance by their dermatologists or other medical doctors about the effectiveness of the treatment and potential side effects. This information should be discussed with both the patient and their family. Therefore, a shared decision-making process that considers all of these factors, along with the risks and benefits of each therapy, is recommended [77]. The best approach involves a personalized and multidisciplinary strategy that involves comprehensive care, potentially involving other professionals, such as psychologists, pulmonologists, allergists, ear–nose–throat specialists, immunologists, paediatricians, nutritionists, gastroenterologists, and psychiatrists. This approach helps optimize treatment by considering not only the patient’s age and disease severity but also a range of disease-related and concomitant factors.

Thus, the most recent approach to treating patients with AD suggests therapy that targets and achieves minimal activity of AD (MDA), where the decision on therapy is based on both patient and clinician decisions [65]. So, for therapy decision and introduction, it is very important to understand and take into account patients’ preferences, as was recorded by many studies (Table 1) [4,84,85,86,87,88,89,90,91,92].

## 8. Conclusions

Overall, the management of patients with AD and decisions regarding systemic therapy are complex, requiring the consideration of multiple factors. Overall, factors to consider before introducing systemic therapy for AD include the severity of AD, treatment efficacy concerns, previous topical therapy and its adverse reactions, patient preferences, age, pregnancy planning, ability and willingness to adhere to the treatment regimen, patient expectations and fears, impact on personal life, intimate aspects, social relationships, work or school performance, patient history and comorbidities, therapeutic preferences, treatment-related risks, and any associated psychological or psychiatric issues. It is very important, therefore, to understand and take into account patients’ preferences and thoughts. According to the latest approach in treating AD patients, it is useful to focus on therapy that targets and achieves minimal activity of AD, where the ultimate decision on therapy is informed by both the patient and the clinician. This shared patient–clinician approach may improve disease management/outcome through increased patient satisfaction with therapy, better patient compliance, and increased engagement. So, in clinical practice, it is recommended that both physicians and patients collaborate in advance to identify specific needs and potential therapeutic goals, leading to an individualized and personalized therapeutic decision through a shared decision-making process and a multidisciplinary approach. This stepwise, interdisciplinary, and personalized approach has shown great value in clinical practice and is supported by studies that have demonstrated improvements in both AD symptoms and related complications.

## Figures and Tables

**Table 1:** Literature data and results from recent studies on atopic dermatitis therapy decisions and patient preferences.

Author, Year	Analysed Factors	Methods and Examinees/Patients	Results	Conclusions
Myers K, et al., 2023 [84]	AD patients’ treatment preferences	Cross-sectional, web-based DCE survey administered to 300 US adults with mild to moderate AD	Most valued treatment outcome: achieving clear skin within 3–4 months. Topical creams applied 2× daily preferred over systemic treatments Respondents with lower self-assessed AD burden were more open to topicals and less concerned about side effects.	→ Findings support shared decision-making in managing mild to moderate AD.
Boeri M, et al., 2022 [85]	Identification of key treatment attributes and preferences for systemic AD treatment	Qualitative interviews with 21 adults with moderate to severe AD; online DCE survey with 320 participants (74% F; mean age 35 yrs)	Top treatment concerns: annual risk of malignancy, mode of administration, probability of clear skin, and time to itch relief. Daily oral treatment preferred over injectable treatment. Higher AE risks accepted for more effective treatments.	→ Findings support joint patient–physician decision-making in managing moderate to severe AD.
Brownstone ND, et al., 2024 [86]	Clinicians’ choices of systemic therapies for AD and PSO without molecular testing, and the frequency of treatment switching	Twenty-question survey assessing treatment strategies for AD and PSO, completed by 265 dermatology conference attendees in 2022	“Reported efficacy” was the top treatment factor, however, 62% of clinicians reported needing ≥2 medications to reach it. A total of 90% found molecular testing useful to improve treatment selection.	→ Molecular tests may help determine the most efficacious drug for individual patients.
Feldman SR, et al., 2024 [90]	Assessment of treatment preferences for those with moderate to severe AD	Online DCE with 300 US adults (70% F) who reported moderate to severe AD or who had tried systemic therapy after finding topical treatments ineffective (June 2023) RI calculated	RI of treatment attributes: itch control (38%), risk of cancer (23%), respiratory infection risk (18%), heart problem risk (11%), sustained improvement in skin appearance (5%), blood test frequency (3%), and frequency and mode of administration (2%). AE attributes accounted for more than half of RI.	→ Treatment efficacy and safety preferred over mode of administration.
Schaarschmidt ML, et al., 2024 [87]	Patient preferences for systemic AD treatments	Online DCE of 182 AD patients in Germany (75.3% F) analysing treatment outcome and process preferences	AEs most important (RIS 31.2), followed by (almost) clear skin (RIS 24.2) and probability of itch improvement (RIS 16.0). Less relevant: application method (RIS 14.4), itch relief onset (RIS 7.4), and lab test frequency (RIS 6.8). Preferences significantly influenced by sex, age, psychiatric comorbidity, current therapy, and HRQOL.	→ Participants prioritize safety and symptom control.
Augustin M, et al., 2020 [4]	Therapeutic needs of patients with AD in routine care	Nationwide cross-sectional study involving 1678 patients (60.5% F) from 91 dermatology practices and outpatient clinics in Germany	High AD burden (mean SCORAD 42.26 ± 18.63, mean DLQI of 8.49 ± 6.45, and mean EQ VAS of 63.62 ± 21.98). ‘Quite important’/‘very important’ patient needs: ‘to be free of itching’ (96.0%), ‘to get better skin quickly’ (87.7%), and ‘to be healed of all lesions’ (85.7%). Treatment needs rated more important by older people, women, and those diagnosed with AD for ≤1 year. Key factors for higher needs: skin-related QoL, greater AD severity, and age.	→ AD patients exhibit diverse therapeutic needs based on individual burdens; identifying these can enhance personalized care and shared decision-making.
Thomas C, et al., 2022 [88]	Patient preferences for AD treatment attributes	Online DCE survey completed by 404 AD patients (65% F) who used AD treatments during the past 2 yrs	Priorities: achieving significant itch reduction and minimizing infection risk. Patients willing to accept lower efficacy for treatments with rapid onset, oral administration, and less frequent check-ups.	→ Understanding patients’ preferences can enhance patient–physician decision-making
Kwatra SG, et al., 2023 [89]	AD patients’ willingness to balance the risks and benefits of systemic treatments	Online DCE survey involving 200 patients with moderate to severe AD, who assessed treatment attribute preferences	Patients prioritized itch reduction, speed of itch relief, and clearing skin and were willing to accept some risk of serious infections and acne for better treatment outcomes.	→ Patients with moderate to severe AD may accept associated risks of systemic treatment. Attention to preferences can enhance patient–physician decision-making
Feldman SR, et al., 2024 [90]	CRI of topical treatments attributes for mild to moderate AD	DCE survey administered to 300 adults and 331 adolescents with AD and 330 caregivers of children with AD in the US	Adults prioritized avoiding skin colour changes (CRI 29.0) and time until itch improvement (26.6). Adolescents less concerned about skin colour changes. Caregivers less concerned about time until clear skin in patients.	→ Physicians should consider age-related differences in treatment preferences.
Ameen M, et al., 2024 [91]	Treatment preferences and priorities of moderate to severe AD patients	Online DCE survey of 713 adults from Denmark, France, the UK, and Canada	Patients prioritized avoiding severe AEs. Daily oral pills preferred over biweekly injections. Less important factors: time to full effect and monitoring.	→ Safety is the highest priority for moderate to severe AD patients, followed by ease of administration.
Okubo Y, et al., 2024 [92]	Patient and physician preferences for new biologic AD treatments	Online DCE survey in Japan involving 323 AD patients and 121 physicians	A total of 46.24% of patients and 76.67% of physicians chose new treatments. Physicians prioritized rash treatment efficacy and cost. Patients favoured add-on therapies and clinic-administered injections.	→ Findings support shared decision-making in clinical practice.

Abbreviations: AD—atopic dermatitis; AE—adverse effect; CRI—conditional relative importance DCE—discrete choice experiment; DLQI—Dermatology Life Quality Index; EQ VAS—EuroQol Visual Analogue Scale; F—female; HRQOL—health-related quality of life; PSO—psoriasis; QoL—Quality of Life; RI—relative importance; RIS—relative importance score; SCORAD—SCORing Atopic Dermatitis; yrs—years.

## Data Availability

The data that support the findings of this study are openly available in PubMed or available in other sources.
